# *In Vitro* Generated Equine Hepatic-Like Progenitor Cells as a Novel Potent Cell Pool for Equine Metabolic Syndrome (EMS) Treatment

**DOI:** 10.1007/s12015-023-10507-3

**Published:** 2023-01-20

**Authors:** Krzysztof Marycz, Nabila Bourebaba, Anna Serwotka-Suszczak, Malwina Mularczyk, Larry Galuppo, Lynda Bourebaba

**Affiliations:** 1 International Institute of Translational Medicine, Jesionowa 11, Malin, 55-114 Wisznia Mała Poland; 2 Department of Surgical and Radiological Sciences, School of Veterinary Medicine, University of California, Davis, Davis, CA 95516 USA; 3 Department of Experimental Biology, Faculty of Biology and Animal Science, Wrocław University of Environmental and Life Sciences, Norwida 27B, 50-375 Wrocław, Poland

**Keywords:** Eq_ASCs, Eq_HPCs, Differentiation, EMS, Liver, IR, PECAM-1, ALB

## Abstract

Equine metabolic syndrome (EMS) is recognized as one of the leading cause of health threatening in veterinary medicine worldwide. Recently, PTP1B inhibition has been proposed as an interesting strategy for liver insulin resistance reversion in both equines and humans, however as being a multifactorial disease, proper management of EMS horses further necessities additional interventional approaches aiming at repairing and restoring liver functions. In this study, we hypothesized that *in vitro* induction of Eq_ASCs hepatogenic differentiation will generate a specialized liver progenitor-like cell population exhibiting similar phenotypic characteristics and regenerative potential as native hepatic progenitor cells. Our obtained data demonstrated that Eq_ASCs-derived liver progenitor cells (Eq_HPCs) displayed typical flattened polygonal morphology with packed fragmented mitochondrial net, lowered mesenchymal CD105 and CD90 surface markers expression, and significant high expression levels of specific hepatic lineage genes including PECAM-1, ALB, AFP and HNF4A. therewith, generated Eq_HPCs exhibited potentiated stemness and pluripotency markers expression (NANOG, SOX-2 and OCT-4). Hence, *in vitro* generation of hepatic progenitor-like cells retaining high differentiation capacity represents a promising new approach for the establishment of cell-based targeted therapies for the restoration of proper liver functions in EMS affected horses.

**Graphical Abstract:**

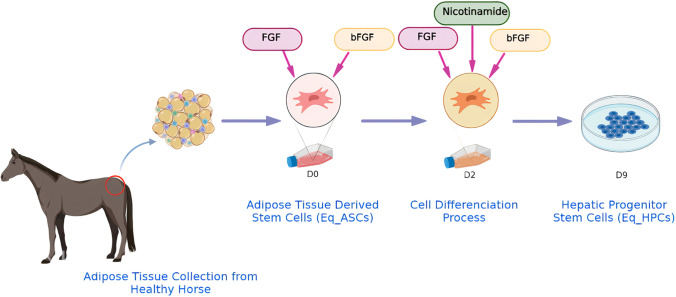

## Introduction

Equine Metabolic Syndrome (EMS) is an increasingly recognized endocrine disorder that is diagnosed in horses, ponies and even donkeys worldwide [1–3]. In fact, despite the behavioral and physiological differences between horses and donkeys; these latter are also believed to be affected by EMS and share similar symptoms, including obesity, insulin dysregulation and laminitis [3]. Laminitis is a common consequence of EMS, characterized by severe foot pain which can have a major impact on the lifestyle and well-being of horses and donkeys, which make it important both from animal welfare and economic points of view [4, 5]. From an etiological point of view, EMS involves a number of clinical symptoms leading to the development of insulin resistance and, as a result, hyperinsulinemia, laminitis, hyperlipidemia, local and systemic inflammation [1, 6]. Although the molecular basis of this condition is not fully understood, it seems that one of the insulin resistance hallmarks is associated to liver metabolism deregulation, what results both in liver fibrosis and hepatitis due to the persistent release of proinflammatory factors by injured hepatocytes [7–9].

One of the potential therapeutic approaches proposed by our group includes inhibition of protein-tyrosine phosphatase PTP1B, which was shown to play a major role in regulating various metabolic and inflammatory mechanisms. In our previous research, we used a low molecular weight inhibitor MSI-1436, that was already shown to improve glucose tolerance and insulin sensitivity in insulin-resistant mice [10–13]. We demonstrated that MSI-1436 not only restores insulin sensitivity, but also modulates underlying molecular events including oxidative stress, mitochondrial biogenesis and ER stress, as a part of its PTP1B inhibitory effect [14, 15]. On the other hand, various studies evidenced the high therapeutic value of stem/progenitor cells-based strategies for the management of metabolic disorders and liver failures [16, 17]. Hepatic progenitor cells are considered as invaluable therapeutic tools for the regeneration of damaged and dysfunctional liver tissue, however, in the course of severe liver injury settings, intrinsic regenerative potential of liver progenitors is compromised, and ineffective for liver homeostasis restoration [18, 19], hence the need for combined and refined therapeutic approaches that will enable to simultaneously regulate liver metabolic biases and maintain high tissue repair rates.

In current research, we proposed to develop a cell-based approach to regenerate insulin-resistant liver using experimentally generated hepatic-like progenitor cells in complement to PTP1B inhibitor application. Since we showed that transplantation of ASCs in EMS horses possesses limited clinical value, we considered the establishment of an equine model of liver progenitor cells (Eq_HPCs), as a novel progenitor cell pool for the treatment of equine metabolic syndrome. In this paper, we present a preliminary study of Eq_HPCs obtained from the guided differentiation of adipose-derived stromal cells (ASCs) along with their phenotypic and morphological characteristics, proliferative potential, as well as the expression of key stemness genes.

## Materials and Methods

The equine adipose tissue-derived stem cells (Eq_ASCs) were obtained from healthy horses’ adipose tissue from three biopsies [20]. The tissues sampling procedures have been approved by the Local Ethics Committee for Animal Experiments in Wroclaw (Resolution no.058/2020, 9.12.2020). Hepatic Progenitor Stem Cells (Eq_HPCs) were obtained following a 9-day differentiation of Eq_ASCs according to the cell differentiation protocol presented by Raquel Taléns-Visconti et al. [21]. Briefly, cells were exposed to Epidermal growth factor (EGF) and Basic fibroblast growth factor (bFGF) from D0 to D2 and then cultured in the presence of bFGF, Nicotinamide and Hepatocyte growth factor (HGF) from D2 to D10. To verify the efficiency of cells’ differentiation, the Eq_ASCs and Eq_HPCs were cultured at different densities to recover sufficient material for the analysis of cell proliferation (MTS Proliferation Colorimetric Assay (Abcam, Cambridge, United-Kingdom)), Population Doubling Time Assay [22], Cells Scratch Assay [23] and MUSE Cell Count Assay (Millipore Muse Count & Viability Kit [24]). Moreover, gene expression analysis (RT-qPCR) [25] (Table 1), protein expression (Western Immunoblot [26] (Table 2), cell surface markers analysis using flow cytometry (FACS) [27] (Table 3), as well as cell morphology (Confocal Microscopy using MitoRed for mitochondria staining and Phalloidin for cytoskeleton staining [28, 29]) were performed on both native Eq_ASCs and differentiated Eq_HPCs. Each analysis was performed in at least three technical repetitions and analysed using GraphPad Prism 8.0.2 (GraphPad Software, San Diego, CA, USA). The statistical differences were calculated using a one-way analysis of variance (ANOVA) and Tukey’s Post-hoc test. The levels of significance were indicated with asterisks: * for *p* < 0.05, ** for *p* < 0.01, and *** for *p* < 0.001. The differences were considered significant with * *p* < 0.05.

**Table 1:** List of the genes

Gene	Primer	Sequence 5’–3’	Amplicon length (bp)	Accession No.
*HNF4A*	F: R:	CAGGAGATGCTGCTGGGAG ATTGTGGTGATGGCTCCTGG	227	XM_003363931.4
*AFP*	F: R:	CAGCCACTTGTTGCCAACTC CTGGCCAACACCAGGGTTTA	125	NM_001081952.1
*KRT18*	F: R:	TGGGGGCCTTACCTCAAGAT CTTTCGGAGCCCATGGATGT	186	XM_005614771.3
*ALB*	F: R:	CTGGTGCTGGTTGCCTTTTC CAGCCAGTTCACCGTAGGTT	202	NM_001082503.1
*Nanog*	F: R:	CCTTAGCTACAAACAGGTTAAGAC TGGTGGTAGGAATAGAGCCC	147	XM_023643093.1
*OCT 4*	F: R:	TCTCTTTGGGAAGGTGTTCAG GTCTCAATACTAGTTCGCTTTCTC	198	XM_023624232.1
*SOX 2*	F: R:	AGAACACCAATCCCGTCCAC TACAAGGTCCATTCCCTCGC	152	XM_023623361.1
*Nestin*	F: R:	ACTGAGAAGTTCCAGCTGGC GAGCGATCCCAATCACACCA	158	XM_023640985.1
*GAPDH*	F: R:	GATGCCCCAATGTTTGTGA AAGCAGGGATGATGTTCTGG	250	NM_001163856.1

*HNF4A*: Hepatocyte Nuclear Factor 4 Alpha; *AFP* Alpha Fetoprotein; *KRT18*: Keratin 18; *ALB*: Albumin; *NANOG*: Nanog Homeobox; *OCT 4*: POU Class 5 Homeobox 1; *SOX 2*: Sex Determining Region Y)-Box 2 ; *Nestin*: Nestin ; *GAPDH*: Glyceraldehyde-3-Phosphate Dehydrogenase

**Table 2:** List of the antibodies for the western blot analysis

Detected protein	Antibody dilution	Catalog no.	mAb Clone	Manufacturer
	Primary antibodies			
PECAM1	1:1000	nbp1-71663	Polyclonal	Novus
β-Actin	1:2500	a5441	AC-15	Sigma-Aldrich/Merck
	Secondary antibodies			
Goat Anti-Rabbit IgG Antibody, Fc, HRP conjugate	1:2500	ap156p	Polyclonal	Sigma-Aldrich/Merck
Anti-Mouse IgG (Fc specific)–Peroxidase antibody produced in goat	1:10000	A0168	Polyclonal	Sigma-Aldrich/Merck

**Table 3:** List of the antibodies for the flow cytometry analysis

Detected protein	Antibody dilution	Catalog no.	mAb Clone	Manufacturer
	Primary antibodies			
PECAM1	1:1000	nbp1-71663	Polyclonal	Novus
Thy1 (CD90)	1:20	555,595	5E10	BD Pharmingen
CD105	1:20	25-1057-42	SN6	Invitrogen
CD45	1:6	555,483	HI30	BD Pharmingen
CD44	1:6	555,479	G44-26	BD Pharmingen
CD34	1:20	21,270,344 × 2	4H11[APG]	Immunotools
	Secondary antibodies			
Alexa Fluor™647	1:200	A21244	Polyclonal	Invitrogen

## Results

### Characterisation of the Equine Hepatic Progenitor Stem Cells (Eq_HPCs) Derived from the Differentiation of the Equine Adipose Tissue Derived Stem Cells (Eq_ASCs)

**Fig. 1:**
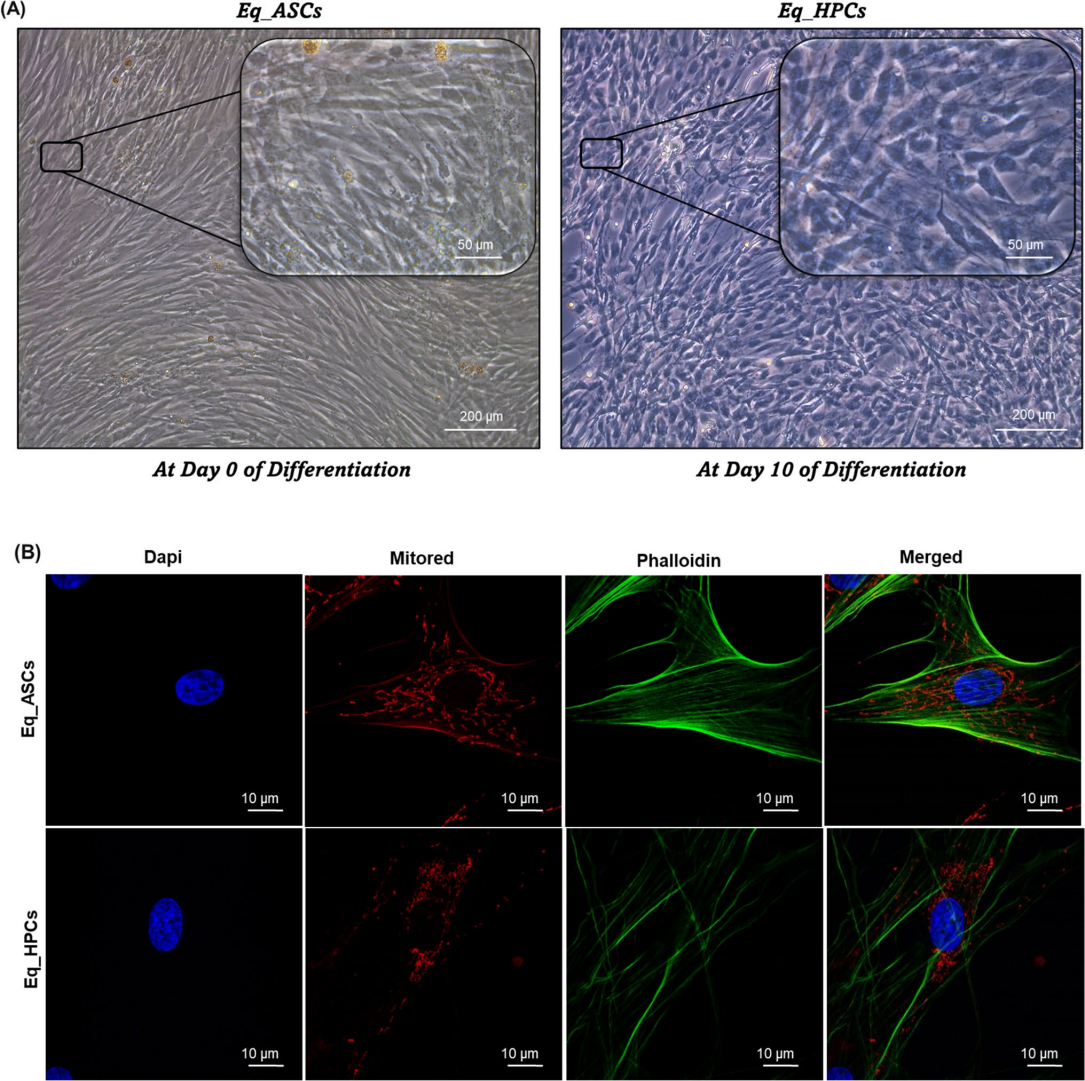

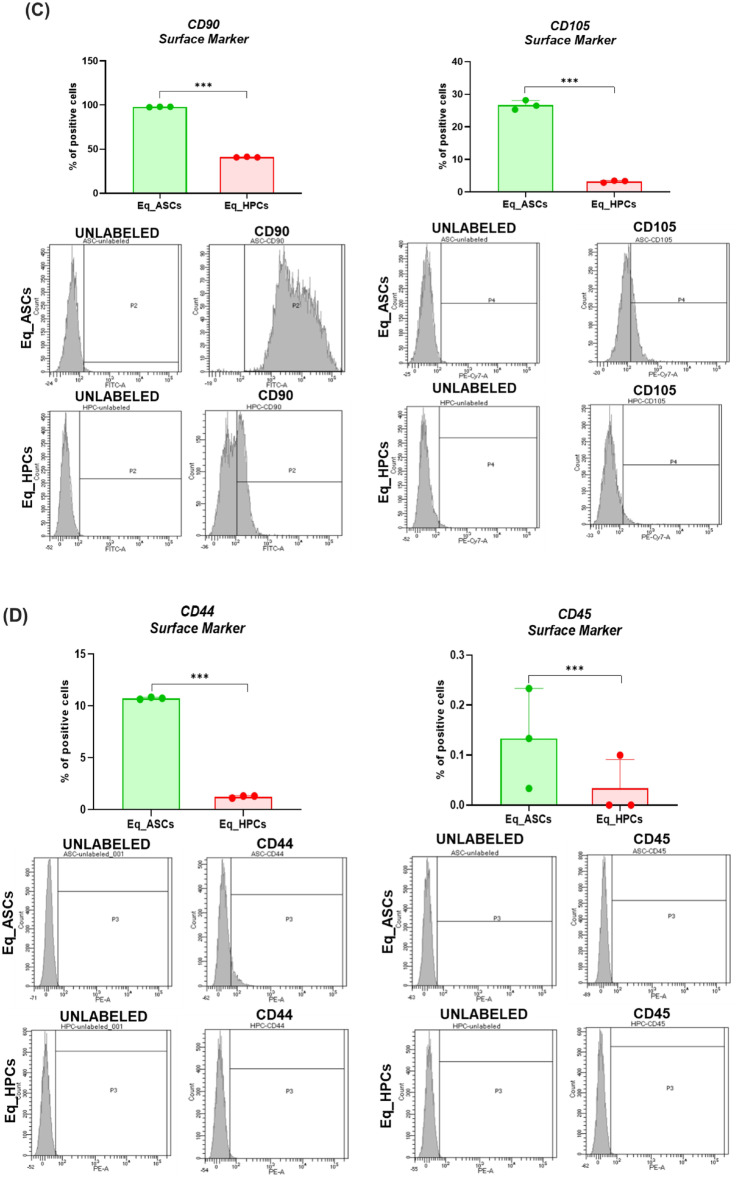

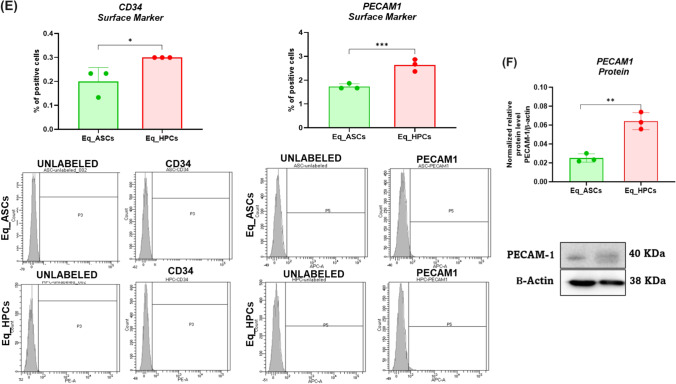
Characterisation of the equine hepatic progenitor stem cells (Eq_HPCs) derived from the differentiation of the equine adipose tissue derived stem cells (Eq_ASCs). **A** Morphology of Eq_ASCs as well as Eq_HPCs. **B** Representative photomicrographs of MitoRed and Phalloidin staining assay obtained by confocal epi-fluorescent microscopy; Bar size 10 μm; magnification × 60. **C** Bar charts depicting the total percentage of CD90 and CD105 cells. **D** Bar charts depicting the total percentage of CD44 and CD45 cells. **E** Bar charts depicting the total percentage of CD34 and CDPECAM1 cells. **F** Relative protein expression of PECAAM1 accompanied with its representative immunoblots. Results were normalized to the expression of endogenous β-actin control. Representative data from three independent experiments are shown ± SD (*n* = 3). An asterisk (*) indicates a comparison of treated group to untreated healthy cells. * *p* < 0.05, ** *p* < 0.01, *** *p* < 0.001. Eq_ASCs: equine adipose tissue derived stem cells; Eq_HPCs: equine hepatic progenitor stem cells

In order to verify the proper differentiation process, a characterization of both Eq_ASCs and Eq_HPCs cells has been assessed via a morphological analysis and flow cytometry test in order to examine cells phenotype by analysing the most relevant markers i.e. CD90, CD105, CD44, CD45, CD34 and PECAM1, as well as a protein profiling of this latter i.e. PECAM1 in order to compare between the two cell type (Fig. 1). As we can observe in the Fig. 1A, the morphological aspect of the cells is different when it comes to the differentiated ones (Eq_HPCs). Likewise, morphologically, Eq_ASCs were fibroblast-like forms typical of stem cells derived from adipose tissue before the start of the cell differentiation process [30]. Subsequently, after 10 days of differentiation, there is a considerable and non-negligible morphological change in the cells; indeed, their appearance tends towards a polygonal oval shape [31] (Fig. 1A). Furthermore, the confocal microscopy inquiry aimed to further confirm these morphological changes by showing the differences in the aspect of mitochondria in both Eq_ASCs and Eq_HPCs cells; indeed, the Fig. 1B revealed that the Eq_ASCs exhibit filamentous appearance and an elongated shape unlike Eq_HPCs which presents tubular and globular mitochondria, which is a specific characteristic of liver cells [32]. Moreover, the percentage of positive cells for both CD90 and CD105 was significantly higher (*p* < 0.001) in Eq_ASCs cells (100% and ± 28% respectively ) when compared to Eq_HPCs cells (< 50% and < 5% respectively) (Fig. 1C). Same results were observed regarding the markers CD44 and CD45; clearly, the percentage of positive cells for both cited markers is also significantly higher (*p* < 0.001) in the Eq_ASCs cells (11% and < 0.15% respectively) compared to Eq_HPCs cells (± 2 and < 0.1% respectively) (Fig. 1D). However, when it comes to the CD34 and PECAM1 markers, the results of the flow cytometry analysis as well as the protein profiling of PECAM1 looks different; indeed, the percentage of positive cells for CD34 and PECAM1 is higher (*p* < 0.05 and *p* < 0.001 respectively) in EqHPCs cells (0.3% and < 3% respectively) compared to Eq_ASCs cells (0.2% and < 2% respectively) (Fig. 1E). Also, the result presented in Fig. 1F confirms the previous result regarding the PECAM-1 marker, in fact, the protein expression of the latter is reduced in Eq_ASCs cells when compared to Eq_HPCs cells (*p* < 0.01) (Fig. 1F).

### Growth Kinetics Evaluation of the Equine Hepatic Progenitor Stem Cells (Eq_HPCs) Derived from the Differentiation of the Equine Adipose Tissue Derived Stem Cells (Eq_ASCs)

**Fig. 2:**
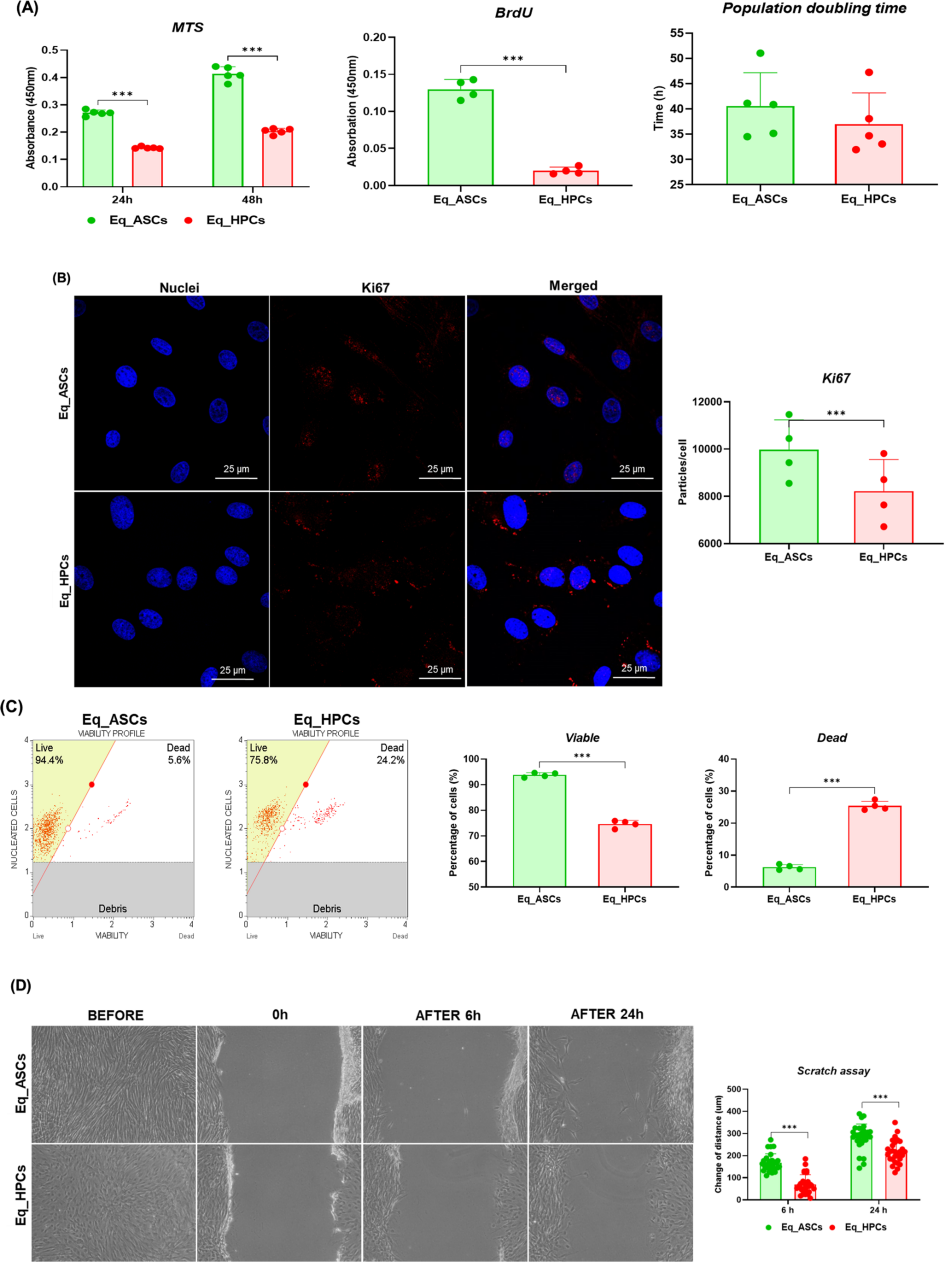
Growth Kinetics Evaluation of the equine hepatic progenitor stem cells (Eq_HPCs) derived from the differentiation of the equine adipose tissue derived stem cells (Eq_ASCs). **A** Histograms represent the average absorbance at 490 nm of MTS tetrazolium; Percentage of incorporated BrdU in newly synthetized DNA; Doubling Population Time estimation based on time in hours. **B** Representative photomicrographs of Ki67 staining assay obtained by confocal epi-fluorescent microscopy; bar size 10 μm; magnification × 60. **C** Representative dot-plots for MUSE Count & Viability assay, and bar-charts depicting the quantitative analysis of live and cell death. **D** Representative micrograph photos of the scratch test taken under an inverse microscope at 0, 6 and 24 h and its representative bar chart for the scratch size. Representative data from three independent experiments are shown ± SD (*n* = 3). An asterisk (*) indicates a comparison of treated group to untreated healthy cells. * *p* < 0.05, ** *p* < 0.01, *** *p* < 0.001. Eq_ASCs: equine adipose tissue derived stem cells; Eq_HPCs: equine hepatic progenitor stem cells

In order to examine the proliferative properties of the equine hepatic progenitor stem cells i.e. Eq_HPCs after the differentiation process (Fig. 2). The cells viability has been analysed using MTS tetrazolium metabolization assay during 24 and 48 h, the results illustrated in Fig. 2A show that Eq_ASCs exhibits a significantly higher (*p > 0.001*) absorbances when compared to the Eq_HPCs cells after 24 and 48 h which correspond to a higher proportion of living Eq_ASCs cells (Fig. 2A). Similar trends were observed after BrdU incorporation analysis, which revealed that Eq_ASCs had increased newly synthetized DNA (*p* < 0.001) and thus improves the proliferative potential, by contrast to Eq_HPCs cells (Fig. 2A). Furthermore, the proportion have been measured in order to determine the population doubling time (Fig. 2A) and the results obtained indicates that the Eq_HPCs display a lower proliferative capacity compared to Eq_ASCs (35 and 40 h respectively). These observations had been supported by the Ki67 staining, which unveil that the proliferative aptitude of the EqASCs is significantly increased when compared to the Eq_HPCs (*p* < 0.001) (Fig. 2B). Moreover, the results obtained from the MUSE analysis of Live/Dead cells exhibits a higher percentage of living cells in for Eq_ASCs, and in parallel, a high rate of dead cells in Eq_HPCs (Fig. 2C). Additionally, the Fig. 2D, represents the results obtained from the scratch assay assessed, we can observe that after 6 h, the diameter of the scratch is more reduced for the Eq_ASCs when compared to the Eq_HPCs (200 μm and < 100 μm respectively) (*p* < 0.001); after 24 h, the size of the scratch is more reduced for both cell type, however, it is still more reduced (*p* < 0.001) for the Eq_ASCs than the Eq_HPCs (< 400 µM and <300 μm respectively) (Fig. 2D).

### Equine Hepatic Progenitor Stem Cells (Eq_HPCs) Derived from the Differentiation of the Equine Adipose Tissue Derived Stem Cells (Eq_ASCs) Stemness

**Fig. 3:**
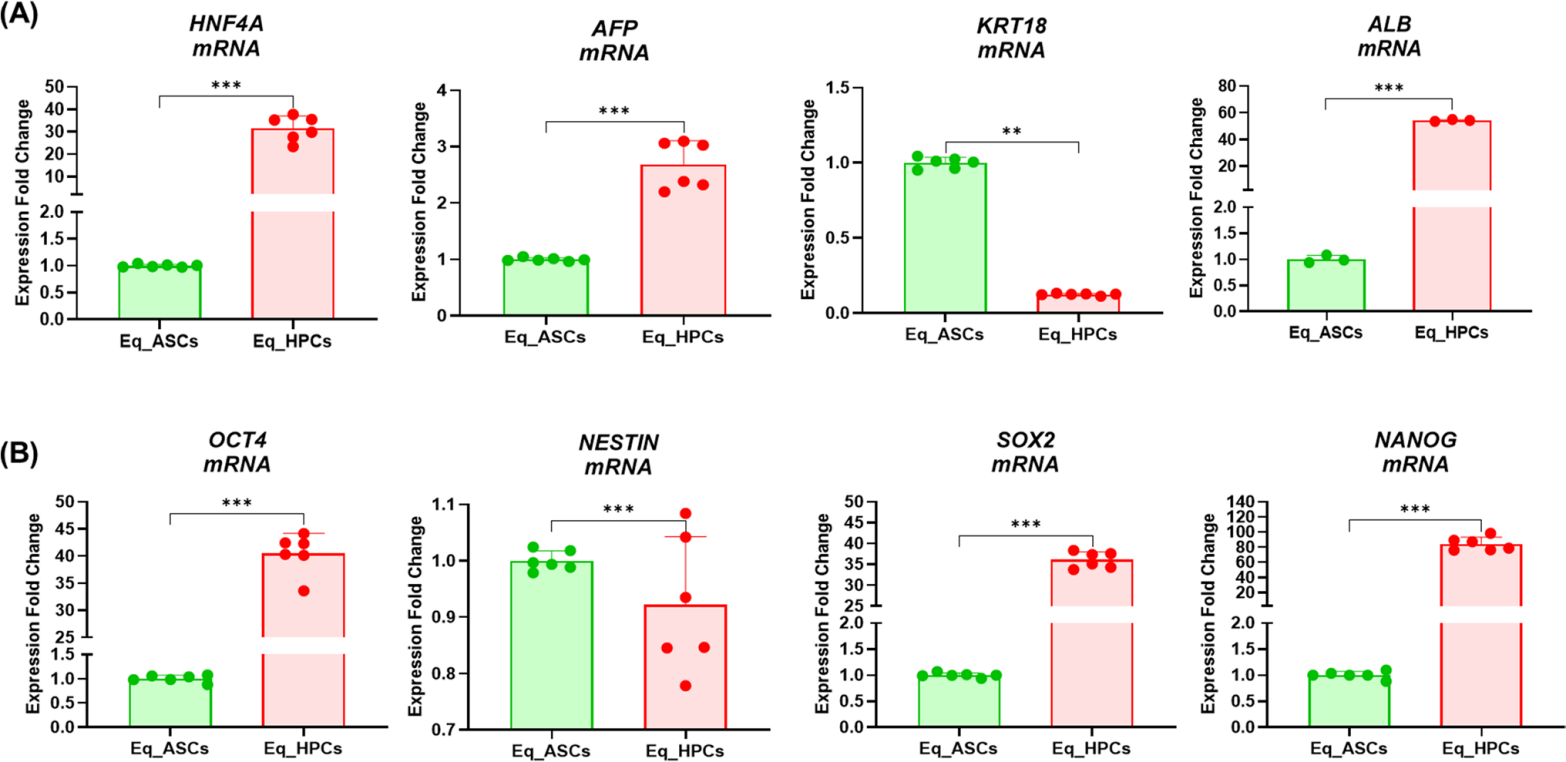
Equine hepatic progenitor stem cells (Eq_HPCs) derived from the differentiation of the equine adipose tissue derived stem cells (Eq_ASCs) Stemness. **A** Relative expression quantitation of *HNF4A*, *AFP*, *KRT18* and *ALB*. **B** Relative expression quantitation of *OCT4*, *NESTIN*, *SOX2* and *NANOG*. Representative data from three independent experiments are shown ± SD (*n* = 3). An asterisk (*) indicates a comparison of treated group to untreated healthy cells. * *p* < 0.05, ** *p* < 0.01, *** *p* < 0.001. Eq_ASCs: equine adipose tissue derived stem cells; Eq_HPCs: equine hepatic progenitor stem cells

The equine hepatic progenitor stem cells’ stemness was evaluated via the analysis of the gene expression of *HNF4A*, *AFP*, *KRT18*, *ALB*, *OCT4*, *NESTIN*, *SOX2* and *NANOG* mRNAs (Fig. 3). The results presented in Fig. 3A, indicates that the Eq_HPCs exhibits a significant elevated expression of *HNF4A*, *AFP* and *ALB* (*p* < 0.001) when compared to the Eq_ASCs cells; however, concerning the *KRT18*, the opposite is observed, the Eq_ASCs present a higher expression of the gene in contrast to the Eq_HPCs (*p* < 0.01) (Fig. 3A). Likewise, the Eq_HPCs display a higher gene expression than the Eq_ASCs (*p* < 0.001) of the *OCT4*, *SOX2* and *NANOG* mRNA. Nevertheless, we note that the genetic expression of the *Nestin* marker is significantly superior in the Eq_ASCs than in the Eq_HPCs (*p* < 0.001) (Fig. 3B).

## Discussion

Liver insulin resistance is an inseparable component of EMS, which is currently one of the most frequent endocrine disorders among horses. One of the potential therapeutic approaches proposed by our group includes inhibition of protein-tyrosine phosphatase PTP1B [14, 15, 33], by systemic application of MSI-1436 and/or systemic administration of equine liver progenitor cells (Eq_HPCs). Here, for the first time, we characterized a model of Eq_HPCs obtained from the guided differentiation of adipose-derived stromal cells (Eq_ASCs), that might serve as a future reliable and accessible therapeutic tool for EMS treatment, and be considered an appropriate alternative to autologous hepatocytes transplantation. Earlier studies reported the possibility to induce *in vitro* differentiation of various human and mouse MSCs populations toward hepatic-like cells and also hepatic progenitor cells, which showed partial to complete hepatic phenotype within 10 to 14 days differentiation and increased expression of ALB, CPM and EPCAM markers [34–38]. In our investigation, obtained Eq_HPCs were characterized by noticeable morphological changes from a polygonal-like structure, typical for hepatic-like cells, by contrast to Eq_ASCs which exhibited common MSCs elongated-fibroblastic morphology. Therewith, confocal microscopy examination evidenced changes in mitochondrial network architecture. Eq_HPCs demonstrated packed short tubular and globular mitochondria, differently from Eq_ASCs in which mitochondria predominantly appeared tubular with spaghetti-like structure. This distinctive mitochondrial organization has been previously attributed to the natural poor fusion capacity of the hepatic mitochondrion, and can be considered as an additional parameter for differentiation efficiency evaluation [39]. The analysis of cell surface markers using FACS technique showed various modifications in the expression patterns of ASCs clusters of differentiation following 10 days of hepatic induction. Eq_HPCs displayed reduced expression of mesenchymal surface markers including CD105, CD90, CD44 and CD45. Furthermore, Eq_HPCs expressed significantly higher levels of the endodermal (Platelet endothelial cell adhesion molecule-1, PECAM-1) in opposition to undifferentiated ASCs. Similarly, obtained Eq_HPCs were found to positively express a defined panel of hepatocyte markers and functionality genes, including albumin (ALB), hepatocyte nuclear factor 4 alpha (HNF4A) and alpha-fetoprotein (AFP), which are important factors regulating hepatic cells proliferation and transcription machinery [40]. Our observed data are in agreement with previous investigations that reported the potential of various human MSCs including ASCs to differentiate into hepatic lineage, and pointed out the characteristic molecular signature of obtained differentiated cells expressing high levels of hepatic progenitor markers ALB, AFP and HNF4A, while losing the mesenchymal-specific CD90 and CD105 markers – all of which, supporting an efficient hepatic specialization [9, 41–43]. Interestingly, the expression of cytokeratin 18 (KRT18), a mature hepatocytes and biliary marker was found to be decreased in Eq_HPCs compared to native Eq_ASCs. This observation stays in line with previous published data of Taléns-Visconti et al. [43], who indicated that expression of both cytokeratin 18 and 19 is neither influenced by the various pro-hepatogenic molecules used for Eq_ASCs differentiation nor critical for proper hepatic specialization. The proliferative potential of progenitor cells is of critical importance for efficient liver regeneration. The evaluation of obtained Eq_HPCs metabolic activity evidenced a reduced viability and proliferation capacity when compared to Eq_ASCs which are native mesenchymal stromal cells with greater survival and expansion capability. However, the detection of high Ki-67 particles, a specific nuclear protein marker for cellular proliferation in Eq_HPCs cultures indicated that the generated progenitors are able to remain proliferative in culture and do not reflect a dormant or quiescent state, which has been similarly reported by Xu and colleagues [44], who demonstrated that Hu_ASCs-derived hepatic-like cells substantially proliferate after engraftment to mice livers.

The ability of liver progenitor cells to differentiate into various hepatic lineages including hepatocytes and cholangiocytes is a prerequisite for their efficient pro-regenerative potential. In this study, we demonstrated that induced hepatogenic differentiation of equine ASCs resulted in liver progenitor-like cells with substantial stemness capacity. Eq_HPCs exhibited upregulated typical stemness markers, i.e., NANOG, SOX-2 and OCT-4 compared to native Eq_ASCs. Our findings are in accordance to previous reports showing that hepatic progenitor cells are enriched in pluripotent markers such as NANOG, SOX2 and OCT-4, which participate in the regenerative and repair properties of the hepatogenic precursors [45].

Taken together, these results further uphold the ability of Eq_ASCs to differentiate into functional and potent liver progenitor-like cells, which shed promising light on the use of *in vitro* model of generated progenitor cells population and their potential therapeutic role in liver regeneration, fibrosis, inflammation and insulin sensitization [45–47].

## Conclusion

This investigation aimed at generating a model of equine liver progenitor-like cells (Eq_HPC) through guided Eq_ASCs hepatogenic differentiation. Obtained data highlighted the high potential of Eq_ASCs to differentiate into hepatogenic precursors characterized by reduced mesenchymal CD105 and CD90 surface markers expression, enriched hepatic lineage PECAM-1, ALB, AFP and HNF4A markers, and enhanced stemness NANOG, SOX-2 and OCT-4 genes. These findings thus provide pledged prospects for the development of new ground-breaking cell-based therapies for the efficient and long-term management of liver failures in the course of equine metabolic syndrome.

## Data Availability

All datasets generated and/or analysed during the current study are presented in the article, the accompanying Source Data or Supplementary Information files, or are available from the corresponding author upon reasonable request.
